# Prehospital management of COPD patients in respiratory failure and short-term outcome

**DOI:** 10.1186/cc12070

**Published:** 2013-03-19

**Authors:** G Campagne, J Cuny, P Gosselin, P Goldstein, N Assez, E Wiel

**Affiliations:** 1 Lille University Hospital Center, Lille Cedex, France

## Introduction

Respiratory failure in COPD patients is a frequent call in French emergency dispatching centers. We have evaluated the prehospital management of COPD patients and severity signs, and analyse outcome in the emergency department or ICU.

## Methods

We conducted an observational, descriptive, retrospective, single-center study during a 4-month period. All COPD patients with respiratory failure and prehospital care were included. Different data were recorded.

## Results

Ninety patients were included (77% male, 23% female). Mean age 69 years (± 11.88). Fifty-five percent were smokers, 52% had arterial hypertension, 39% received long-term oxygenotherapy, 18% received antibiotics in the 7 days before, 18% corticosteroids, and 14% were on long-term NIV support at home. An emergency medical ambulance was immediately sent for 86% of patients. Ninety-two percent had normal consciousness (Glasgow Coma Scale 15), 78% had bronchospasm, 71% had signs of respiratory struggle, and 12% were unable to speak. The mean respiratory rate was 31.4 cycles/ minute (± 8.18), the average cardiac pulse was 103.6 beats/minute (± 23.14). Nasal EtCO_2 _44.92 mmHg (± 16.38), pulse oximetry with air was 83.48% (± 12.09), and the average flow rate of oxygen delivered was 5.69 l/minute (± 2.93). None of the patients had fever. Eighty-five percent were supported on spontaneous ventilation, 22% received prehospital non-invasive ventilation, they all showed signs of severity and 3% need tracheal intubation. Seventy-five percent of patients received β_2_-agonist and anticholinergic nebulization, 45% intravenous corticosteroids. Seventy-one percent were admitted to the emergency room, 29% to the ICU.

## Conclusion

Most of the patients had signs of severity and bronchospasm. The absence of fever and antibiotics allows us to think that the cause of decompensation is not pneumonia. Although most of them were hypoxic and hypercapnic, they seem to be good candidates for NIV support in prehospital care. Very few studies report the use of NIV in cases of COPD respiratory failure in the first care delivered at home.
